# Regulatory interplay between SR proteins governs *CLK1* kinase splice variants production

**DOI:** 10.1261/rna.080107.124

**Published:** 2024-12

**Authors:** Lulzim Shkreta, Aurélie Delannoy, Johanne Toutant, Benoit Chabot

**Affiliations:** RNA group, Department of Microbiology and Infectious Diseases, Faculty of Medicine and Health Sciences, Université de Sherbrooke, Sherbrooke, Quebec, Canada J1E 4K8

**Keywords:** CLK kinases, SR proteins, alternative splicing, dCas13Rx, phosphorylation

## Abstract

The CLK1 kinase phosphorylates SR proteins to modulate their splicing regulatory activity. Skipping of alternative exon 4 on the *CLK1* pre-mRNA produces a CLK1 variant lacking the catalytic site. Here, we aimed to understand how various SR proteins integrate into the regulatory program that controls *CLK1* exon 4 splicing. Previously, we observed that the depletion of SRSF10 promoted the inclusion of *CLK1* exon 4. Using the expression of tagged proteins and CRISPR/Cas9-mediated knockouts in HCT116 cells, we now identify TRA2β, TRA2α, SRSF4, SRSF5, SRSF7, SRSF8, and SRSF9 as activators of exon 4 inclusion. In contrast, SRSF3, SRSF10, and SRSF12 elicit exon 4 skipping. Using CRISPR/dCas13Rx and RNA immunoprecipitation assays, we map an enhancer in exon 4 interacting with TRA2β. Notably, CLK1 kinase inhibitors antagonized the repressor activity of HA-SRSF10, HA-SRSF12, and HA-SRSF3. Our results suggest that *CLK1* exon 4 inclusion is determined primarily by a balance between the activities of TRA2 proteins and CLK-phosphorylated SRSF3. CLK-phosphorylated SRSF10 and SRSF12 would interact with TRA2 proteins to prevent their enhancer activity, allowing SRSF3 to enforce exon 4 skipping more efficiently. Our study provides insight into the complex regulatory network controlling the alternative splicing of *CLK1*, which uses CLK1-mediated phosphorylation of SR proteins to regulate the inclusion of catalytic exon 4 in *CLK1* transcripts.

## INTRODUCTION

Alternative splicing is part of the expression program of nearly all human pre-messenger RNAs (pre-mRNAs) (Pan et al. 2008; Wang et al. 2008). Although alternative splicing is used to generate variants with distinct functions, it can control protein level by producing noncoding variants that get degraded by nonsense-mediated RNA decay (NMD) (Wollerton et al. 2004; Leclair et al. 2020; Reixachs-Sole and Eyras 2022). Alternative splicing is integral to the normal regulatory program of many biological processes (Blencowe 2006). On the other hand, alternative splicing is often altered in human diseases, and splicing dysregulation has been observed in every hallmark of cancer (Shkreta et al. 2013; Chabot and Shkreta 2016; Bradley and Anczukow 2023). The mechanisms by which splicing dysregulation occurs in diseases vary considerably, from somatic mutations that directly affect the splicing of key genes to the altered expression of splicing regulatory proteins (Dvinge et al. 2016). Important players that are contributing to regulatory programs include SR and hnRNP proteins. Recent work suggests that their combinatorial contributions may rest on their existence as part of complexes (Damianov et al. 2016; Gueroussov et al. 2017; Zhou et al. 2021). Additional layers of control are offered by transcription coupling, as well as by signaling, allowing to link splicing decisions to environmental cues and metabolic needs.

The phosphorylation of SR proteins can have a variety of outcomes on SR protein function and activity. In vitro phosphorylation of recombinant SRSF1 enhanced binding to U1 snRNP 70K protein, and is required for SRSF1 activity in splicing in vitro (Xiao and Manley 1997). Hyperphosphorylation of SRSF1 occurs upon DNA damage (Leva et al. 2012). Phosphorylation of SRSF2 regulates its RNA-binding protein solubility and oligomerization (Kundinger et al. 2021). Based on its phosphorylation status, SRSF10 can act either as a positive or a negative splicing factor; dephosphorylated SRSF10 (dSRSF10) blocks splicing, while its phosphorylation converts it into a splicing activator (Feng et al. 2008). The role of SRSF10 phosphorylation in alternative splicing is likely more complex. There are potentially dozens of amino acids in SRSF10 that can be phosphorylated, and their combinatorial arrangement may confer distinct functions. For example, mutating serines 131 and 133 affect interactions with hnRNP F/H and K to impact *Bcl-x* splicing (Shkreta et al. 2016). Mutating other residues like threonine 255 compromises interaction with 14-3-3 proteins, and their dissociation elicits further dephosphorylation of SRSF10 (Shi and Manley 2007).

The CLK, SRPK, and DYRK families of kinases phosphorylate SR proteins (Nayler et al. 1997; Duncan et al. 1998; Wang et al. 1998; Alvarez et al. 2007; Shi et al. 2008; Qian et al. 2011; Ding et al. 2012; Yin et al. 2012; Zhou et al. 2012; Tam et al. 2020). Other proteins that can phosphorylate SR proteins include PRPF4 (Kojima et al. 2001) and protein kinase A (Chen et al. 2014). The mostly cytoplasmic SRPKs can intervene in the nucleus where they bind to CLK1, enhancing its kinase activity, and also acting as a release factor for SRSF1 (Aubol et al. 2016, 2018). The CLKs family includes four members (CLK1–4). CLK1 and CLK4 are considered almost identical, whereas CLK3 is most distantly related. While SRPKs primarily target the RS domain, CLKs phosphorylate SR proteins at specific serine residues within the hinge region and RS domain. CLK1 has been implicated in several processes, from viral RNA maturation to cancer (Babu et al. 2020; Dahal et al. 2021, 2022; Pastor et al. 2021; ElHady et al. 2023). Unlike SRPKs, which generally promote splicing activity, CLK-mediated phosphorylation can have both stimulatory and inhibitory effects on SR protein function, depending on the specific SR protein and the target pre-mRNA. Thus, the phosphorylation of SR proteins can have diverse effects on their activity, influencing subcellular localization, interaction with pre-mRNA, and overall splicing behavior.

CLK1 activity is regulated by the alternative splicing of exon 4 whose skipping produces a protein lacking kinase activity (Uzor et al. 2018; Menegay et al. 2000). The production of an mRNA encoding an active kinase can be restored by treatments that induce SR proteins dephosphorylation, such as heat shock, osmotic stress, or the use of CLK inhibitors (Ninomiya et al. 2011). *CLK1* alternative splicing is also regulated by temperature and circadian rhythms (Haltenhof et al. 2020; Meinke et al. 2020; Neumann et al. 2020). The above studies suggest that the control of alternative splicing of *CLK1* pre-mRNA is used to modulate CLK1 activity, and to ensure rapid rephosphorylation of splicing factors following a stress signal.

A previous study has shown that SRSF10 promotes the skipping of *CLK1* exon 4 (Sohail et al. 2021). However, the precise interplay between SRSF10 and other SR proteins in this regulatory program remains unexplored. Our study delves into the network of SR proteins governing *CLK1* exon 4 splicing. We use a multifaceted approach that includes the expression of HA-tagged proteins, CRISPR/Cas9-mediated knockouts, RNA immunoprecipitation assays, and drug treatments to understand the role of various SR proteins. Our findings reveal a suite of SR proteins functioning as activators or repressors of *CLK1* exon 4 inclusion. We identify SRSF3 as a master repressor of exon 4 inclusion, while SRSF10 and SRSF12 antagonize the function of activators represented by TRA2, SRSF4, SRSF7, SRSF8, and SRSF9. Importantly, our data indicate that the contribution of SRSF3, SRSF10, and SRSF12 to exon 4 inclusion is modulated by their phosphorylation by the CLK kinases, thereby highlighting the feedback mechanisms that govern the alternative splicing of *CLK1*.

## RESULTS

### Impact of SR proteins on *CLK1* exon 4 splicing

**FIGURE 1.**
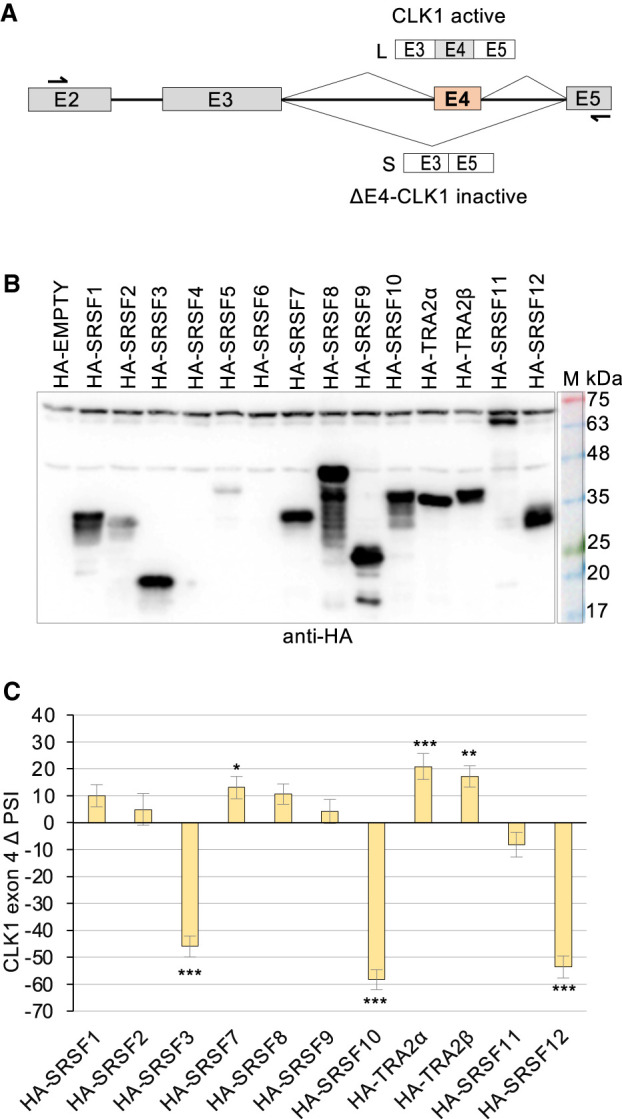
Modulation of *CLK1* exon 4 splicing by tagged SR proteins. (*A*) Representation of the alternative splicing unit of *CLK1* exon 4. The position of primers used for end point RT-PCR is indicated. (*B*) HCT116 cells transfected with the HA-SR plasmids were tested for HA-SR expression by immunoblotting with an anti-HA antibody. (*C*) End point RT-PCR was carried out to monitor endogenous *CLK1* exon 4 inclusion. PSI values obtained for each sample were compared with the HA-EMPTY plasmid to obtain ΔPSI values, which are represented in the graph. Statistical significance was evaluated using multiple *t*-test analysis (GraphPad Prism software, version 10.2.2). (*) *P* < 0.05, (**) *P* < 0.01, and (***) *P* < 0.001. Gel triplicates for each sample displaying the RT-PCR reactions are shown in Supplemental Figure S1A.

Given that CLK1 is a kinase that phosphorylates SR proteins, important connections may be revealed by investigating whether SR proteins can regulate *CLK1* splicing. To assess the role of the major SR proteins in *CLK1* exon 4 splicing, we first expressed 14 HA-tagged SR proteins in HCT116 cells and monitored endogenous *CLK1* pre-mRNA splicing by end point RT-PCR (Fig. 1A). The expression of the tagged proteins was confirmed by immunoblots (Fig. 1B). We noted weak or no expression for HA-SRSF2, HA-SRSF4, HA-SRSF5, and HA-SRSF6. HA-TRA2α, HA-TRA2β, and HA-SRSF7 promoted the inclusion of *CLK1* exon 4 with a difference in percent splice index (ΔPSI) between HA-SR and HA-EMPTY >10. In contrast, HA-SRSF3, HA-SRSF10, and HA-SRSF12 repressed exon 4 inclusion (ΔPSI >40) (Fig. 1C; Supplemental Fig. S1). A repressor role for SRSF3 in *CLK1* splicing is consistent with previous reports that showed that digitoxin and digoxin deplete the levels of SRSF3 to promote *CLK1* exon 4 inclusion (Anderson et al. 2012; Liu et al. 2013). The impact of TRA2β, SRSF3, and SRSF10 was reproduced with different tags (Myc-TRA2β, Myc-SRSF3, and FLAG-SRSF10) (Supplemental Fig. S2). In addition, Myc-SRSF4 and His-SRSF9 promoted exon 4 inclusion in HCT116 cells. No effect was detected with Myc-SRSF2 and Myc-Tra2α.

**FIGURE 2.**
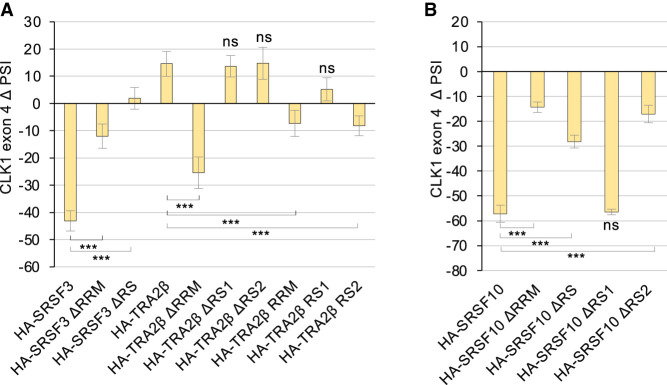
Modulation of *CLK1* exon 4 splicing by domains of SRSF3, TRA2β, and SRSF10. Plasmids allowing the expression of HA-tagged SRSF3 and TRA2β (*A*) or SRSF10 (*B*) proteins or portions thereof were transfected and tested for their impact on endogenous *CLK1* exon 4 splicing. End point RT-PCR reactions were carried out to monitor *CLK1* exon 4 inclusion. PSI values obtained for each sample were compared with the HA-EMPTY plasmid to obtain ΔPSI values, which are represented in the graph. Statistical significance was evaluated using multiple *t*-test analysis. (*) *P* < 0.05, (**) *P* < 0.01, and (***) *P* < 0.001; (ns) not significant. Gel duplicates for each sample displaying the RT-PCR reactions are shown in Supplemental Figure S1B.

To determine which domains of TRA2β, SRSF3, and SRSF10 were required for activity, we expressed derivatives in HCT116 cells (Supplemental Fig. S3). Keeping either the RS1 or the RS2 domain of HA-TRA2β provided full activity but removing both domains abrogated function (Fig. 2A; Supplemental Fig. S1B). A HA**-**TRA2β derivative lacking the RRM domain (TRA2βΔRRM) behaves as a dominant negative and promoted *CLK1* exon 4 skipping. Thus, a free RS domain of TRA2β may sequester other proteins that are part of the activator complex. Removing the RRM or RS portion of HA-SRSFR3 nearly completely abolished activity (Fig. 2A; Supplemental Fig. S1B). HA-SRSF10ΔRRM was essentially inactive, while the activity of HA-SRSF10ΔRS was severely compromised (Fig. 2B; Supplemental Fig. S1B). Notably, the removal of the RS1 portion did not affect the activity of SRSF10, while the removal of the RS2 portion did (Fig. 2B; Supplemental Fig. S1B).

**FIGURE 3.**
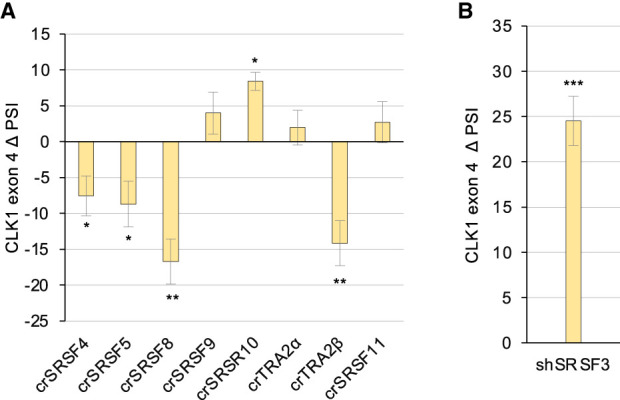
Impact of depleting SR proteins on *CLK1* exon 4 splicing. (*A*) HCT116 cells were transfected with a collection of plasmids to promote CRISPR/Cas9 modification of genes encoding various SR proteins. Following extensive validation by DNA sequencing, quantitative RT-PCR and immunoblots, cell lines that did not express the cognate SR proteins were tested for *CLK1* exon 4 splicing by end point RT-PCR analysis. PSI value for each sample was obtained and compared with a derivative HCT116 cell line made with an empty vector to produce the ΔPSI values for *CLK1* exon 4. (*B*) We used shRNA-expressing and shSCRAMBLE adenovirus vectors to deplete SRSF3 in HCT116 cells. The ΔPSI value is indicated in the graph. Statistical significance was evaluated using multiple *t*-test analysis. (*) *P* < 0.05, (**) *P* < 0.01, and (***) *P* < 0.001. Replicates for each sample displaying the RT-PCR reactions are shown in Supplemental Figure S1C.

**FIGURE 4.**
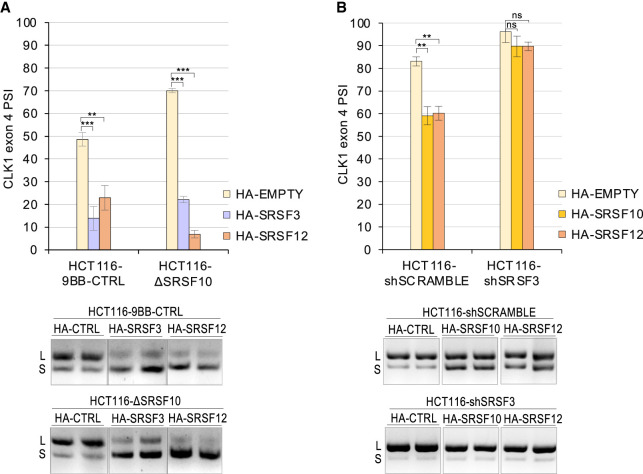
SRSF10 is not required for the activity of SRSF3 and SRSF12, but SRSF3 is required for the activity of SRSF10 and SRSF12. (*A*) HCT116ΔSRSF10 cells and a control HCT116 cell line were used to test the activity of HA-SRSF3 and HA-SRSF12. Forty-eight hours after transfection with the HA-SR or a control (EMPTY) plasmids, RNA was extracted, and RT-PCR assays were conducted to monitor *CLK1* exon 4 splicing (duplicates shown). PSI values were compiled and represented in a graph according to cell lines. (*B*) HCT116 cells were infected with an shSRSF3 or shSCRAMBLE adenovirus and then transfected with HA-SR or control (EMPTY) plasmids. Samples were processed as in *A*.

Because overexpressing proteins or derivatives can lead to indirect effects caused by the sequestering of other regulatory factors, we aimed to confirm the activity of SR proteins by generating HCT116 cell lines in which the expression of individual SR proteins was abrogated by CRISPR-mediated knockouts. For knockouts that could not be obtained or confirmed, we attempted siRNA or shRNA expression. Successful CRISPR/Cas9-mediated knockouts were obtained for SRSF4, SRSF5, SRSF8, SRSF9, SRSF10, SRSF11, TRA2α, and TRA2β (Supplemental Fig. S4A). The depletion of SRSF4, SRSF5, SRSF8, and TRA2β reduced *CLK1* exon 4 inclusion (Fig. 3A; Supplemental Fig. S1C), indicating their role as activators. Notably, the depletion of TRA2α had no significant impact, in contrast to what was anticipated based on the activity of HA-TRA2α. This may indicate that TRA2β can functionally compensate for the loss of TRA2α. The depletion of SRSF10 led to a small increase in exon 4 inclusion (Fig. 3A; Supplemental Fig. S1C; larger increases were detected in other experiments, see Fig. 4A), consistent with the repressor activity for HA-SRSF10.

siRNAs allowed to significantly reduce the expression of SRSF1, SRSF6, and SRSF7 in HCT116 cells (Supplemental Fig. S4B). However, these reductions were not associated with a shift in *CLK1* splicing (Supplemental Fig. S4C). An adenovirus-derived vector carrying an inducible shSRSF3 partially depleted SRSF3 in HCT116 cells (Supplemental Fig. S4D) and promoted exon 4 inclusion (Fig. 3B; Supplemental Fig. S1C), consistent with the repressor activity of HA-SRSF3. Due to poor antibody and inactive siRNAs, we could not confirm the repressor activity of SRSF12 by the various depletion strategies.

Overall, the CRISPR/Cas9 depletion assay, the siRNA-mediated assay, and the HA-SR expression assay led to a somewhat consistent picture of SR proteins-based regulation of *CLK1* exon 4 splicing in HCT116 cells, with SRSF3, SRSF10, and possibly SRSF12, acting as repressors and TRA2β acting as the more potent activator (Table 1).

**TABLE 1.**
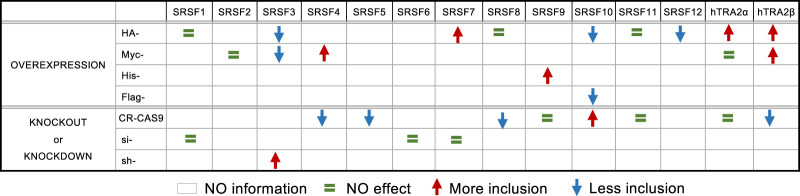
Summary of the impact of expressing tagged SR proteins and depleting endogenous SR proteins on *CLK1* exon 4 splicing.

Splicing regulators often exist as part of complexes (Damianov et al. 2016; Gueroussov et al. 2017; Zhou et al. 2021). To examine the functional links between some of the splicing regulators of *CLK1* exon 4 splicing, we tested how the depletion of SRSF10 affected the repressor activity of SRSF3 and SRSF12. In HCT116 cells depleted of SRSF10 (HCT116ΔSRSF10), the repressor activity of HA-SRSF3 and HA-SRSF12 remained strong (Fig. 4A), possibly indicating that these factors are not part of a common repressor complex. The ability of SRSF12 to repress inclusion was significantly stronger when SRSF10 was depleted, possibly indicating that SRSF12 may be functionally homologous to SRSF10, and thus that a drop in SRSF10 might allow SRSF12 to display a compensatory behavior. In contrast, in HCT116 cells partially depleted of SRSF3, the repressor activity of HA-SRSF10 and HA-SRSF12 was severely compromised (Fig. 4B), indicating that the SRSF3 is important for SRSF10 and SRSF12 to elicit exon 4 skipping.

We also tested the impact of depleting TRA2β on the ability of HA-SRSF10 and HA-SRSF12 to promote exon skipping. While in HCT116 cells partially depleted of TRA2β (HCT116ΔTRA2β) exon 4 inclusion was reduced, the repressor activities of HA-SRSF10 and HA-SRSF12 were still present, but the amplitude of their effect was not significantly affected (data not shown).

### *CLK1* exon 4 contains a splicing enhancer interacting with TRA2β

**FIGURE 5.**
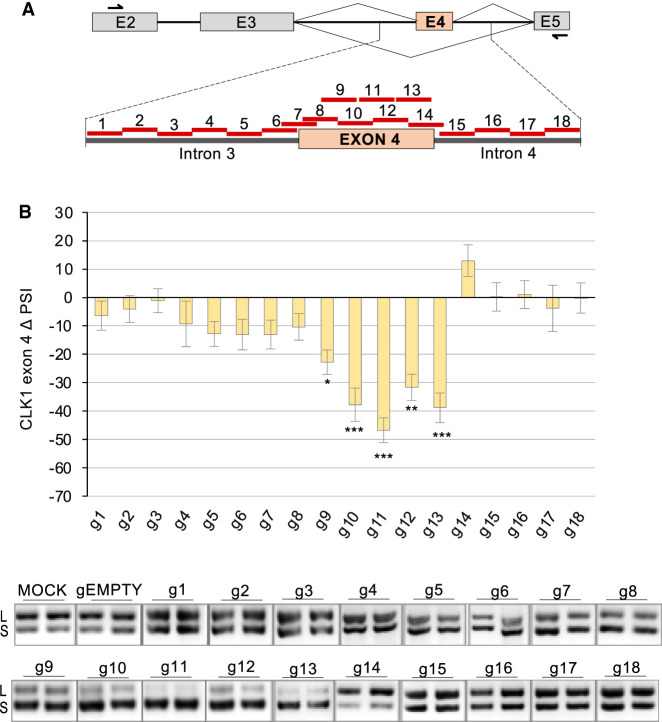
Impact of gRNAs/dCas13Rx on *CLK1* exon 4 splicing in HCT116 cells. (*A*) The positions on the *CLK1* exon 4 alternative splicing unit of 25 nt-long gRNAs tested are indicated. The position of primers used for the RT-PCR is also shown. (*B*) Following transfection of the plasmids expressing the gRNA and dCas13Rx, RNA was extracted, and *CLK1* exon 4 splicing was monitored by RT-PCR. (*B*) ΔPSI values that compare the impact of each guide relative to the gEMPTY plasmid are shown. Duplicates of the RT-PCR products fractionated on acrylamide gels are shown *below* the graph. The statistical significance of values was evaluated using multiple *t*-test analysis of technical triplicates. (*) *P* < 0.05, (**) *P* < 0.01, and (***) *P* < 0.001.

Next, we inquired about the existence of regulatory *cis*-acting elements in and around *CLK1* alternative exon 4. For that purpose, we used an RNA-targeting catalytically inactive dCas13Rx (dCasRx) to sterically block the binding of potential regulatory factors (Konermann et al. 2018; Leclair et al. 2020). A set of approximately twenty 24–26 nt-long dCasRx guide RNAs (gRNAs) was used to target sequences on *CLK1* exon 4 and surrounding intron regions (Fig. 5A). As monitored by end point RT-PCR, most exon-targeting gRNAs promoted dCasRx-mediated exon skipping (g9–g13) consistent with the presence of an enhancer in *CLK1* exon 4 (Fig. 5B), although we cannot rule out that exon 4-containing transcripts are destabilized by gRNAs that target the exon. A similar result was obtained in EcR293 cells (Supplemental Fig. S5). gRNAs g4, g5, g7, and g8, which target the 3′ss/branchsite region of *CLK1* exon 4, also promoted skipping in EcR293 cells. Our results are therefore consistent with the presence of a large enhancer region in *CLK1* exon 4.

**FIGURE 6.**
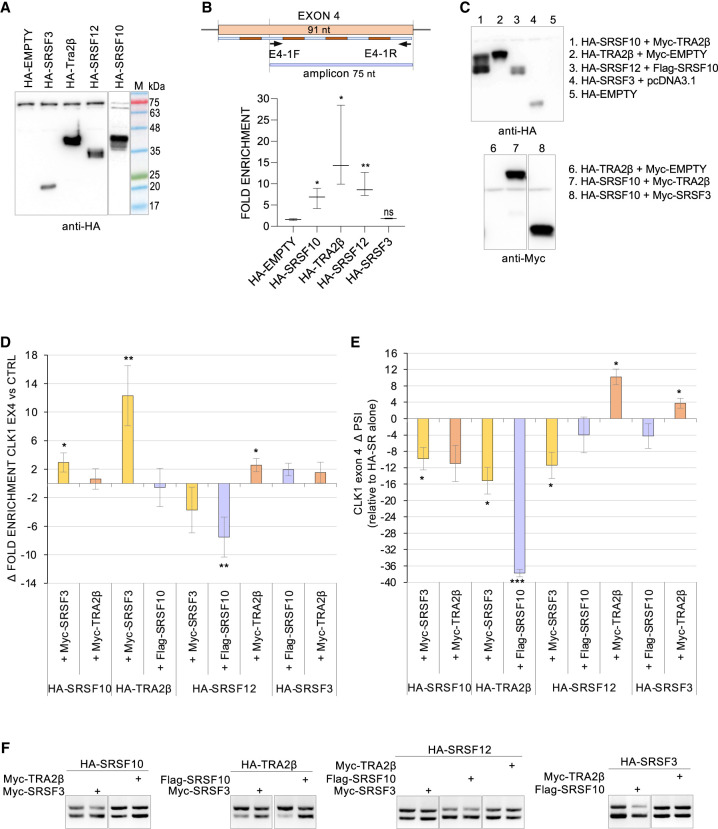
Interaction of HA-SR proteins with *CLK1* exon 4. RNA immunoprecipitation was carried out on cells transfected with the indicated HA-SR and an HA-EMPTY plasmids. (*A*) The expression of HA-tagged proteins was validated by immunoblotting an anti-HA antibody. (*B*) The recovered RNA was quantitated for *CLK1* exon 4 using primers shown. The differential between values obtained for each sample is plotted as fold enrichment of recovery. Raw data for RNA immunoprecipitations are provided in Supplemental Table S1. (*C*) Dual expression of differentially tagged SR proteins as determined by immunoblotting with the relevant antibodies. (*D*) Impact of the dual expression on the recovery of exon 4 sequences. (*E*) Impact of dual expression of SR proteins on *CLK1* exon 4 splicing. Values plotted correspond to ΔPSI that compare the shift in exon 4 splicing of the dual combination to the shift obtained with each HA protein, and thus the capacity of the Myc-SR or FLAG-SR protein to change the impact of the HA-SR protein alone. Duplicates of RT-PCR reactions are shown *below* (panel *F*). The statistical significance of values was evaluated using multiple *t*-test analysis. (*) *P* < 0.05, (**) *P* < 0.01, and (***) *P* < 0.001.

To obtain information on the potential interaction of SRSF10, SRSF12, SRSF3, and TRA2β on *CLK1* exon 4, we performed RNA immunoprecipitations using anti-HA antibody and HCT116 cells expressing tagged proteins (Fig. 6A). Quantitative RT-PCR was used to assess recovery of the main portion of *CLK1* exon 4 (Fig. 6B). While we assume that SR proteins primarily interact with exon 4 during the pre-mRNA stage, we cannot exclude the possibility of interactions occurring when *CLK1* is partially or fully spliced. HA-TRA2β was the protein that displayed the strongest recovery of exon 4 sequences. HA-SRSF12 and HA-SRSF10 displayed an intermediate interaction ability, while no recovery was obtained with HA-SRSF3. To examine the interplay between these factors, we expressed combinations of them and performed RNA immunoprecipitations using the anti-HA antibody (Fig. 6C,D). We monitored how the expression of a Myc-SR or a FLAG-SR changed the interaction of a HA-SR with exon 4. FLAG-SRSF10 antagonized the interaction of HA-SRSF12 with exon 4 without affecting exon 4 inclusion (Fig. 6E,F), a result that would be consistent with functional redundancy. While FLAG-SRSF10 did not affect HA-TRA2β binding (Fig. 6D), it strongly neutralized its ability to promote exon inclusion (Fig. 6E,F), possibly indicating that SRSF10 might neutralize TRA2β activity on the enhancer. In contrast, Myc-SRSF3, which interfered with the activity of HA-TRA2β, significantly increased its interaction with exon 4 (Fig. 6D–F). In this case, splicing inhibition by SRSF3 may possibly lead to an accumulation TRA2β bound to unspliced exon 4. These results suggest that the mechanisms by which SRSF3 and SRSF10 implement repression are distinct. Myc-TRA2β and Myc-SRSF3 have little impact on the binding of HA-SRSF10 and HA-SRSF12 (Fig. 6D).

### Inhibiting CLK kinases impairs the exon-4 repressing activity of SRSF10, SRSF12, and SRSF3

**FIGURE 7.**
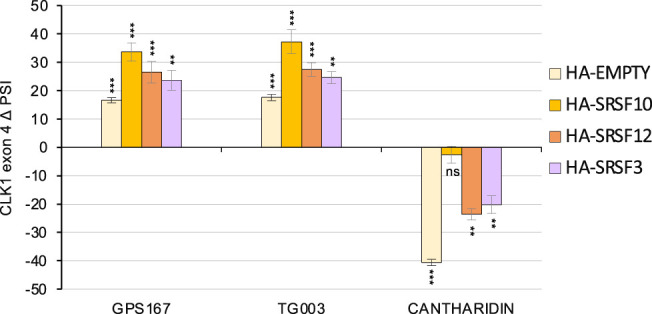
Impact of drugs affecting phosphorylation on the activity of SR proteins that repress *CLK1* exon 4 inclusion. HCT116 cells transfected with plasmids allowing the expression of HA-SRSF10, HA-SRSF12, HA-SRSF3 or with an HA-EMPTY control plasmid were treated with DMSO, GPS167, TG003, and cantharidin. RNA was extracted after 48 h and analyzed by RT-PCR for *CLK1* exon 4 inclusion. The ΔPSI values are plotted in the graph. Triplicates RT-PCR reactions fractionated on gels are shown in Supplemental Figure S6. (**) *P* < 0.01 and (***) *P* < 0.001, (n.s) not significant.

Given that CLK1 phosphorylates SR proteins, it would make biological sense for *CLK1* exon 4 splicing to be sensitive to the phosphorylation status of SR proteins that we have identified as regulating *CLK1* splicing. Given that SRSF10 is possibly the weakest substrate for the CLK kinases (Shi and Manley 2007), a slight drop in CLK1 kinase activity in the cell would therefore be expected to first impact SRSF10 phosphorylation. SRSF10 phosphorylation status could therefore be used as a sensor to regulate *CLK1* splicing; a phosphorylated SRSF10 (SRSF10p) may promote exon skipping to decrease the level of active CLK1. The CLK kinases inhibitor GPS167 dephosphorylates SRSF10 and promotes the inclusion of *CLK1* exon 4 inclusion (Fig. 7; Supplemental Fig. S6; Sohail et al. 2021). TG003 is another CLK kinases inhibitor (Sakuma et al. 2015) and it had a similar impact on exon 4 splicing (Fig. 7). In addition, both GPS167 and TG003 antagonized the impact of HA-SRSF10, HA-SRSF12, and HA-SRSF3 on exon 4 (Fig. 7), consistent with the notion that their dephosphorylation abrogates their repressor activity. In agreement with this model, cantharidine, an inhibitor of the PP1 phosphatase that dephosphorylates SRSF10 and other SR proteins (Shi and Manley 2007), promoted *CLK1* exon 4 skipping (Fig. 7; Supplemental Fig. S6). Moreover, cantharidine improved the ability of HA-SRSF12 and HA-SRSF3 to promote exon 4 skipping but did not affect that of HA-SRSF10, possibly because SRSF10 may already be fully phosphorylated. Our results, therefore, support the view that the phosphorylation of SRSF10, SRSF12, and SRSF3 enforces *CLK1* exon 4 skipping.

**FIGURE 8.**
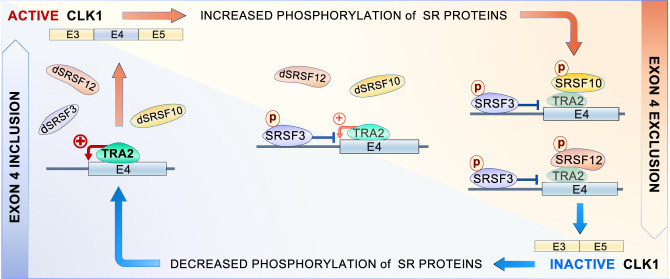
Schematic model of the interplay between SRSF3, SRSF10, SRSF12, and TRA2 proteins in the control of *CLK1* exon 4 splicing. When the demand for active CLK1 is high (e.g., when SRSF3, SRSF10, and SRSF12 are dephosphorylated [i.e., dSRSFR3, dSRSF10, and dSRSF12]), exon 4 inclusion would rely on TRA2 proteins to act through the exonic enhancer. The existence of phosphorylated SRSF3 (but not of phosphorylated SRSF12 or SRSF10) would create a competition between TRA2-mediated activation and SRSF3-mediated repression, here occurring at the 3′ splice site of exon 4, to produce intermediate levels of active CLK1. Phosphorylated SRSF10 and SRSF12 would associate with TRA2 proteins to interfere with the enhancer activity, allowing SRSF3 to repress exon 4 inclusion more efficiently.

Overall, our results can be reconciled into a simple model for the regulation of *CLK1* exon 4 inclusion in HCT116 cells (Fig. 8). The TRA2 proteins would be part of an activator complex binding to the enhancer of *CLK1* exon 4. Increased expression of TRA2 proteins would favor enhancer complex assembly. The TRA2-mediated increase in exon 4 inclusion would be antagonized by phosphorylated SRSF3 (SRSF3p). On the other hand, phosphorylated SRSF10 and SRSF12 (SRSF10p and SRSF12p) would block the activity of the TRA2-containing activator complex. SRSF10 has been documented to interact with TRA2β (Shkreta et al. 2017), and the interaction of SRSF10 with TRA2α is lost when SRSF10 is dephosphorylated (Shin et al. 2004). A similar scenario may occur with SRSF12p. Thus, the interaction of SRSF10p or SRSF12p with TRA2 may abrogate the ability of the activator complex to offset the impact of SRSF3, leading to more efficient repression. The TRA2/SRSF10 interaction may be stabilized by SRSF10 binding to exon 4 since the RRM domain of SRSF10 is important for its repressor activity on exon 4. Thus, a weak CLK1 substrate like SRSF10 (and probably also SRSF12) may help fine-tune the production of active CLK1: an important drop in CLK1 activity would lift SRSF3p-, SRSF10p-, and SRSF12p-mediated repression, whereas a smaller drop may only lead to the dephosphorylation of SRSF10p and SRSF12p. While this model offers a framework for understanding the intricate interactions among splicing proteins in regulating *CLK1* exon 4 inclusion, it is important to emphasize that our comprehension of the compensatory mechanisms remains incomplete. Specifically, the interplay between TRA2 proteins and other positive regulators, as well as the interactions between the negative regulatory proteins SRSF10 and SRSF12, require further experimental validation. Future studies should focus on elucidating these complex relationships to provide a more comprehensive and accurate picture of the regulatory process.

## DISCUSSION

We have investigated how SR proteins control the alternative splicing of *CLK1* exon 4 in HCT116 cells. The interference assay using gRNAs bound by dCas13Rx identified a large enhancer region in *CLK1* exon 4. Given that HA-TRA2β increased exon 4 inclusion, that the CRISPR/Cas9-mediated knockout of TRA2β gave the opposite effect, and that TRA2β interacts with exon 4, TRA2β is most likely promoting enhancer activity. In contrast, while HA-TRA2α was similarly active, the knockout of TRA2α did not affect *CLK1* exon 4 splicing. These results suggest that while TRA2α may not be required for normal enhancer activity, it may display functional redundancy with TRA2β. Myc-SRSF4, HA-SRSF7, and His-SRSF9 also promoted moderate levels of exon 4 inclusion, and the knockout of SRSF4, SRSF5, and SRSF8 elicited exon 4 skipping. These results may point to the existence of a single enhancer complex made up of multiple factors bound to exon 4. Alternatively, a range of individual but redundant activities may contribute to *CLK1* exon 4 inclusion, either by competing with TRA2β for access to the enhancer element or by binding to distinct portions of exon 4.

While we did not identify silencer elements in or immediately surrounding *CLK1* exon 4, HA-SRSF10, HA-SRSF12, and HA-SRSF3 repressed exon 4 inclusion, a result confirmed by the impact of depleting SRSF10 and SRSF3. This apparent partnership between SRSF10 and SRSF3 in the regulation of *CLK1* exon 4 skipping distinguishes it from a study reporting SRSF10 and SRSF3 as acting in opposite manners on alternative exons carrying m^6^A modifications (Xiao et al. 2016). As for SRSF12, it was initially identified as capable of antagonizing the activity of other splicing factors in vitro and modulating alternative splicing in vivo (Cowper et al. 2001). Notably, the repressor activities of HA-SRSF10 and HA-SRSF12 were compromised when SRSF3 was depleted. In contrast, the repressor activity of HA-SRSF3 remained strong when SRSF10 or SRSF12 was depleted. These results led us to propose that SRSF3 is the master repressor, while SRSF10 and SRSF12 act as corepressors by antagonizing TRA2β (Fig. 8). SRSF3 was previously shown to repress *CLK1* exon 4 inclusion in WI38 cells (Liu et al. 2013). The reactivity of *CLK1* exon 4 to digoxin, a drug that inhibits the expression of SRSF3 (Anderson et al. 2012), suggested the presence of SRSF3 binding sites in an internal region of intron 3 (Liu et al. 2013) that was not covered by our gRNAs. Thus, SRSF3 may interact in the upstream intron to repress the 3′ splice site of *CLK1* exon 4 by a mechanism that remains to be determined.

HA-SRSF10 expressed in the presence of the CLK1 inhibitors TG003 and GPS167 was considerably less active at promoting *CLK1* exon 4 skipping. Given that the phosphorylation of SRSF10 increases its interaction with TRA2 proteins (Shin et al. 2004), we propose that SRSF10p neutralizes TRA2-mediated enhancer activity (Fig. 8). Likewise, because SRSF12 represses exon 4 inclusion, that SRSF10 antagonizes the interaction of SRSF12 with exon 4 without impacting exon 4 inclusion, and that the activity of SRSF12 is compromised by CLK inhibitors, SRSF10p and SRSF12p may independently abrogate TRA2 function. Thus, the phosphorylation status of SRSF10 and SRSF12 may act as a sensor for CLK activity, allowing to determine how much active CLK1 kinase is required. Using SRSF10 as a sensor makes homeostatic sense since SRSF10 is a weak substrate for CLK kinases (Shi and Manley 2007), and thus possibly one of the first SR proteins affected by a small drop in CLK activity. A dSRSF10 (and possibly dSRSF12 as well) would therefore rapidly feedback on exon 4 splicing to allow the production of a functional CLK1 kinase (Fig. 8).

The importance of phosphorylation on SRSF10 activity is well documented. SRSF10 behaves as a general splicing activator when phosphorylated, and a repressor when dephosphorylated (Shin and Manley 2002; Shin et al. 2004; Feng et al. 2008). The role of SRSF10 phosphorylation in alternative splicing appears more complex. For example, the splicing impact of oxaliplatin-mediated SRSF10 dephosphorylation was transcript-specific since oxaliplatin co-opted SRSF10 into controlling the alternative splicing of transcripts not normally subjected to SRSF10 regulation, while in other cases, it converted SRSF10 from acting as a repressor of exon inclusion into an activator (Shkreta et al. 2016; Cloutier et al. 2018). Notably, the RS2 domain of SRSF10 was essential to control *CLK1* exon 4 splicing, while the RS1 domain was dispensable. A reverse requirement was noted for *Bcl-x* splicing (Shkreta et al. 2016). While the RS1 and RS2 domains share common functional features, the phosphorylation status of multiple residues in these domains may modulate the interaction with splicing regulators whose identity may vary with different transcripts. Thus, the effects of SR protein phosphorylation on alternative splicing are likely to be complex and context-dependent.

In summary, our results reveal a complex interplay between multiple SR proteins in regulating the alternative splicing of *CLK1* exon 4. TRA2β appears to act as a key activator. Other SR proteins like TRA2α, SRSF4, SRSF5, SRSF7, SRSF8, and SRSF9 may also contribute to enhancer activity, possibly to help fine-tune the production of active CLK1 in response to specific needs. In contrast, SRSF3, SRSF10, and SRSF12 act as repressors in a phosphorylation-dependent manner, allowing homeostatic feedback control of *CLK1* splicing. While our model highlights a molecular scenario that explains how homeostasis of SR protein phosphorylation may be achieved through *CLK1* splicing regulation, it is important to note that our study has been limited to the HCT116 colorectal cancer cell line. Whether a similar process operates in other cancer cells and in a normal cellular context remains to be investigated.

## MATERIALS AND METHODS

### Cell culture and plasmids

HCT116 cells (ATCC) and EcR-293 cells (Invitrogen) were cultured in the recommended medium supplemented with 10% fetal bovine serum (FBS) at 37°C in a humidified atmosphere with 5% CO_2_. Plasmids expressing HA-SRSF10, related derivatives, and FLAG-SRSF10 have been previously described (Shkreta et al. 2016). The EMPTY vector contained the HA-tag but no coding sequence (CDS). Plasmids expressing HA-tagged SRSF1, SRSF2, SRSF3, SRSF4, SRSF5, SRSF6, SRSF7, SRSF8, SRSF9, SRSF10, SRSF11, SRSF12, TRA2α, TRA2β, SRSF3-mutants, and TRA2β-derivatives were previously described (Leclair et al. 2020).

### CRISPR/Cas9 knockouts, siRNA-, shRNA-mediated knockdown, and CRISPR/dCas13Rx assays

HCT116 cells with CRISPR/Cas9-mediated knockout of SR proteins were produced by transfecting the plasmid pSpCas9(BB)-2A-Puro carrying the gRNA sequence along with Cas9. The sequences of gRNAs cloned into the BbsI digested pSpCas9(BB)-2A-Puro vector are shown in Supplemental Table S2. The integrity of the plasmids was verified by sequencing. After 24 h, HCT116 cells transfected either with the plasmid pSpCas9(BB)-empty or plasmids expressing specific SR protein-specific gRNA were selected for puromycin resistance. Selected cells were pulled, grown, and frozen at low passages. Knockouts of SR protein expression were confirmed either by immunoblots or PCR and sequencing of genomic DNA isolated from selected HCT116 cell populations. Sequencing results were analyzed by the TIDE online software (Brinkman et al. 2014). Knockdowns of SRSF1, SRSF6, and SRSF7 were performed using ON-TARGETplus siRNA from Dharmacon (SRSF1 Cat#J-018672-09; SRSF6 Cat#J-016067-09; SRSF7 Cat#J-015909-05). Adenoviruses expressing shRNA for silencing SRSF3 or shScramble control were obtained from Pr. Mannix Auger-Messier at Université de Sherbrooke, and sequences are shown in Supplemental Table S2.

For the CRISPR/dCas13Rx assay, the specific gRNA targeting sequences of *CLK1* exon 4 and surrounding intron regions (shown in Supplemental Table S2) were prepared by annealing forward and reverse 24–26 nt-long DNA oligos synthesized by IDT (Integrated DNA Technologies, Inc.) and subsequently cloned into the BbsI digested pCR8-Cas13d-DR-ccdB gRNA plasmid (Leclair et al. 2020). The pAC1801-pmax-dCasRx plasmid encoded the RNA-targeting catalytically inactive RxCas13d (dCasRx) (Du et al. 2020).

### Transfections

Transfection or cotransfection of cells with expression plasmids or siRNAs (final concentration of 100 nM) was performed using Lipofectamine 2000 (Invitrogen) according to the manufacturer's instructions. For adenovirus infection, we used an MOI (multiplicity of infection) of 50 for shSRSF3- and shScramble-adenoviruses.

### Immunoblot analysis

Cells were harvested, washed with PBS, and whole-cell extracts were prepared by lysing cells in Laemmli sample buffer. Total proteins were quantified using the Lowry method. Equal amounts of total protein were fractionated on SDS-PAGE gels, and standard blotting protocols were carried out for transferring proteins onto nitrocellulose membranes. The membranes were blocked with TBS-T + 5% milk for 1 h, incubated overnight at 4°C with the primary antibody (1:1000 in TBS-T + 2% milk), and for 1 h with horseradish peroxidase-coupled secondary antibodies (1:5000 in TBS-T + 5% milk). The signal was revealed using the Clarity Western ECL substrate (Bio-Rad Cat#1705061), and image acquisition was performed with the Azure 280 imager (Azure Biosystems).

### RNA extraction and RT-PCR analysis

Total RNA from harvested cells was extracted using TRIzol reagent (Invitrogen) according to the manufacturer's instructions. The splicing profile of endogenous *CLK1* was assessed by RT-PCR. In total, 500–1000 ng of RNA was reverse transcribed using OmniScript Reverse Transcriptase (Qiagen) with a mixture of oligo(dT) and random hexamers. One-tenth of the cDNA material was used as a template for the PCR reaction with Taq DNA polymerase and primers listed in Supplemental Table S2. PCR products were separated on an agarose gel, stained with RedSafe Nucleic Acid Staining Solution (FroggaBio), and imaged using the GelDoc Imaging System (MBI Lab Equipment). The intensity of the bands was quantified using ImageJ software, and the percent spliced-in (PSI) ratio of each exon-containing transcript was calculated as the exon-included product intensity divided by the sum intensity of exon-included and exon-excluded products. ΔPSI was calculated as PSI_case_ – PSI_control_ (HA-EMPTY vector for minigene experiments or EMPTY-gRNA for dCAS13Rx experiments).

### RNA immunoprecipitation assays

HCT116 cells transfected or cotransfected with plasmids expressing HA-, Myc-, or FLAG-tagged SR proteins or tag-only control plasmids were harvested 48 h posttransfection. After washing with PBS, cell pellets were resuspended into NET2 buffer supplemented with protease and RNase inhibitors and lysed by sonication. The insoluble material was removed by centrifugation at 4°C. An aliquot of cleared cell lysates was used as the input aliquot, while equal aliquots of the remaining material were immunoprecipitated using either anti-HA-Magnetic beads (MedChemExpress, HY-K0201) or IgG-coupled-Magnetic beads (SureBeads, Bio-Rad Labs). For RNA extraction, after three washing cycles of beads with NET2 buffer, the TRIzol reagent was added directly onto beads pellets. RNA extracted from input, HA-IP and IgG-IP samples was quantified and purity assessed. Equal quantities of RNA from each sample were reverse transcribed using Omniscript RT kit (Qiagen) and the random primer mix. qPCR reactions, in triplicates for the cDNA of each sample, were used as templates for Supermix 2X qPCR (SYBR Green) (Plateforme de Purification des Protéines, Université de Sherbrooke), and a set of primers targeting *CLK1* were done on QuantStudioTM 3 SmartStart Thermocycler (Thermo Fisher). To determine the relative abundance of *CLK1* transcripts in immunoprecipitated samples, the validated Ct values (Ct conf < 0.98) were compared using the Ct-input (preimmunoprecipitated) as the reference, while the difference between HA-IP and IgG-IP control was calculated using the of 2^−ΔΔCt^ method and expressed as fold enrichment.

### Statistical analysis

All experiments presented were performed in technical triplicates. The statistical analysis and the assessment of differences relative to controls were performed using GraphPad Prism software (version 10.2.2).

## SUPPLEMENTAL MATERIAL

Supplemental material is available for this article.
